# Association of pulmonary rehabilitation outcomes with inflammatory and nutritional status in patients with chronic respiratory diseases: a real-world retrospective cohort study

**DOI:** 10.3389/fmed.2026.1876212

**Published:** 2026-09-23

**Authors:** Jingcheng Dong, Shan Jiang, Maimaititusun Yalikun

**Affiliations:** Department of Integrative Medicine, Huashan Hospital, Fudan University, Jing'an, China

**Keywords:** chronic respiratory disease, C-reactive protein, forced expiratory volume in one second, nutritional status, pulmonary rehabilitation, retrospective cohort study, six-minute walk distance

## Abstract

**Objective:**

This study aimed to investigate the interrelationships among pulmonary rehabilitation outcomes, systemic inflammation, and nutritional status in patients with chronic respiratory diseases, and to provide evidence supporting—but not establishing causality for—multidimensional clinical intervention strategies.

**Methods:**

A real-world retrospective cohort analysis was performed on clinical records of 100 patients with chronic respiratory diseases hospitalized in the Department of Respiratory Medicine of a tertiary respiratory center in China between January 2018 and December 2022. Pre- and post-comprehensive rehabilitation (pharmacotherapy, pulmonary rehabilitation exercise, individualized nutritional support, and health education) data were collected, including pulmonary function parameters (FEV1, FVC, FEV1/FVC, TLC, and RV), six-minute walk distance (6MWD), inflammatory markers (C-reactive protein, white blood cell count, and neutrophil percentage), and nutritional indicators (body mass index, serum albumin, and total protein). Cases with >20% missing data were excluded; remaining missing values were handled by complete case analysis. Paired t-tests were used to evaluate pre-post differences, multiple linear regression to identify independent predictors of pulmonary function improvement, and Pearson correlation analysis to examine inter-variable associations. Regression covariates were selected by univariate screening at *p* < 0.20.

**Results:**

The mean patient age was 57.74 ± 8.81 years, with 41 males (41%). Following intervention, FEV1 increased from 1.47 ± 0.43 L to 1.65 ± 0.44 L (*p* < 0.001), 6MWD improved from 428.97 ± 88.59 m to 470.40 ± 92.99 m (*p* < 0.001), and BMI rose from 18.15 ± 2.40 to 18.59 ± 2.47 kg/m^2^ (*p* < 0.001). CRP decreased from 6.85 ± 4.50 to 4.95 ± 4.71 mg/L (*p* < 0.001). Seventy-six percent of patients exceeded the FEV1 minimal clinically important difference (MCID, ≥100 mL), and 63% exceeded the 6MWD MCID (≥30 m). Correlation analysis demonstrated a moderate positive association between ΔFEV1 and Δ6MWD (*r* = 0.41, *p* < 0.001) and a significant negative association between ΔFEV1 and ΔCRP (*r* = −0.21, *p* = 0.039). Multiple linear regression showed that no variable independently predicted ΔFEV1 after full adjustment (overall model *p* = 0.297); the nutritional adequacy score showed only a non-significant trend (*β* = 0.024, *p* = 0.093), interpreted as an exploratory finding.

**Conclusion:**

Comprehensive intervention was associated with significant improvements in pulmonary function, exercise tolerance, and nutritional status alongside reductions in systemic inflammation in patients with chronic respiratory diseases. The observed correlations between changes in FEV1, 6MWD, and CRP highlight potentially interrelated mechanisms, though causality cannot be established from this retrospective, uncontrolled design. These findings support multidimensional management strategies, pending validation in prospective controlled trials.

## 1 Introduction

Chronic respiratory diseases, encompassing chronic obstructive pulmonary disease (COPD), bronchiectasis, and interstitial lung disease (ILD), represent a major component of the global disease burden, contributing substantially to morbidity and mortality worldwide (1). According to the Global Burden of Disease Study, COPD ranks as the third leading cause of death globally, and is projected to affect over 400 million individuals by 2030 (2). Accumulating evidence indicates that these conditions extend beyond localized airway or parenchymal damage, involving systemic inflammation and multi-organ dysfunction (3).

Although the present cohort includes bronchiectasis and ILD patients alongside COPD, the pathophysiological evidence base, particularly concerning systemic inflammation and nutritional interactions, is most developed for COPD and therefore anchors much of the mechanistic discussion below. Decline in pulmonary function constitutes a hallmark of these diseases, with forced expiratory volume in one second (FEV1) serving as the standard metric for assessing airflow obstruction severity and disease progression (4). However, pulmonary function deterioration is not solely attributable to pulmonary pathology; it is also closely linked to systemic inflammatory burden and nutritional status (5).

C-reactive protein (CRP), a sensitive marker of systemic inflammation, has been associated with increased risk of COPD exacerbations and elevated mortality (6). Similarly, white blood cell (WBC) count and neutrophil percentage reflect inflammatory activity and play important roles in disease monitoring.

Malnutrition and impaired nutritional status more broadly are prevalent among patients with chronic respiratory diseases, with reported rates ranging from 20 to 70% (7). Body mass index (BMI), serum albumin, and total protein are commonly used for nutritional assessment. Impaired nutritional status can lead to respiratory muscle atrophy and diaphragmatic weakness, thereby compromising ventilatory capacity and exercise tolerance (8). Furthermore, it impairs immune function and increases susceptibility to infections, creating a detrimental cycle (9). Recent evidence suggests that nutritional intervention can be associated with improved pulmonary function and quality of life in COPD patients (10).

The six-minute walk test (6MWT) is a practical tool for evaluating exercise capacity and overall functional status; the six-minute walk distance (6MWD) correlates strongly with daily activity levels, quality of life, and prognosis (11). Improvement in 6MWD is frequently regarded as a key outcome in comprehensive pulmonary rehabilitation programs.

Although previous studies have separately examined the relationships between inflammation or nutrition and pulmonary function, a systematic investigation of the integrated associations among pulmonary function improvement, inflammatory changes, and nutritional status across diverse chronic respiratory diseases remains warranted. This study retrospectively analyzed clinical data from 100 patients to elucidate the associations among pulmonary function, inflammatory response, and nutritional status, to evaluate the patterns of change following comprehensive intervention, and to explore potential predictors of pulmonary function improvement, while acknowledging the inherent limitations of an uncontrolled retrospective design.

## 2 Materials and methods

### 2.1 Study design and patient selection

This single-center, real-world retrospective cohort study was approved by the Ethics Committee of Changsha Wangcheng District People’s Hospital (a tertiary referral hospital), Hunan Province, China (Approval No.: 2022-IRB-156; approval date: 15 June 2022). The study was conducted at a tertiary referral hospital with a dedicated respiratory medicine department, serving a mixed urban–rural catchment population. As the analysis used de-identified retrospective data, the requirement for individual written informed consent was waived by the ethics committee. Patients hospitalized in the Department of Respiratory Medicine between January 2018 and December 2022 who completed at least six months of follow-up were eligible for inclusion.

#### 2.1.1 Inclusion criteria

(1) Age 18–80 years; (2) confirmed diagnosis of COPD, bronchiectasis, or ILD based on international guidelines (1) (,2), clinical symptoms, pulmonary function testing, and high-resolution computed tomography; (3) disease duration of at least six months; (4) receipt of standard pharmacotherapy, pulmonary rehabilitation, and nutritional support during hospitalization; (5) completion of pulmonary function assessment, 6MWT, and laboratory testing at both baseline and follow-up; (6) complete clinical records.

#### 2.1.2 Exclusion criteria

(1) Major thoracic or abdominal surgery within three months; (2) active malignancy; (3) severe heart failure (NYHA class III–IV); (4) chronic renal failure (eGFR < 30 mL/min/1.73 m^2^); (5) severe hepatic insufficiency (Child-Pugh class C); (6) uncontrolled acute infection; (7) psychiatric or cognitive impairment precluding cooperation; (8) pregnancy or lactation; (9) missing data exceeding 20%.

#### 2.1.3 Missing data handling

Patients with more than 20% missing values across measured variables were excluded per the exclusion criteria above. For the remaining enrolled patients, complete case analysis was applied for regression modeling, as the proportion of sporadically missing values was low (< 5% per variable). No multiple imputation was performed given the small volume of missing data.

#### 2.1.4 Confounding control

Given the observational, uncontrolled design, confounding was addressed analytically by including key prognostic covariates (age, sex, disease type, disease duration, baseline FEV1, BMI, and albumin) in all multivariable models. Residual confounding from unmeasured factors (comorbidity severity, socioeconomic status, medication adherence) cannot be excluded and represents a recognized limitation.

### 2.2 Intervention protocol

All patients received a comprehensive treatment regimen comprising: (1) Pharmacotherapy: disease-specific medications including bronchodilators, inhaled corticosteroids, long-acting β2-agonists, or long-acting muscarinic antagonists as clinically indicated (12); (2) Pulmonary rehabilitation: inspiratory muscle training (30–60% of maximal inspiratory pressure) (13), aerobic exercise (3–5 sessions/week, 30–45 min/session at 60–80% of maximal heart rate), and breathing exercises (14); (3) Nutritional support: individualized plans providing high-protein diets (1.2–1.5 g/kg/day), appropriate carbohydrate intake, and essential fatty acids, with enteral supplementation as necessary (15); (4) Health education: smoking cessation counseling, disease self-management, and medication adherence guidance.

### 2.3 Clinical data collection

Data were retrospectively extracted from electronic medical records.

#### 2.3.1 Baseline characteristics

Age, sex, disease duration, disease type, smoking history, comorbidities, prior viral infections, and history of malnutrition.

#### 2.3.2 Definition of malnutrition history

History of malnutrition was determined by review of pre-admission medical records using the Global Leadership Initiative on Malnutrition (GLIM) framework (16). A patient was classified as having a history of malnutrition if, in records preceding the current admission, they met at least one phenotypic criterion (BMI < 18.5 kg/m^2^ for adults < 70 years or < 20 kg/m^2^ for adults ≥ 70 years; unintentional weight loss > 5% within 6 months or > 10% beyond 6 months; reduced muscle mass documented clinically) combined with at least one etiologic criterion (reduced food intake ≤ 50% of energy requirements for > 1 week, or documented chronic inflammatory disease). All classifications were independently re-verified by two investigators (with disagreements resolved by consensus).

#### 2.3.3 Pulmonary function parameters

Spirometry was performed using the Jaeger MasterScreen system (CareFusion, Germany) in accordance with ATS/ERS standards (17). FEV1, FVC, FEV1/FVC, TLC, and RV were recorded. Each parameter was measured at least three times, with the best value selected. Testing was conducted by experienced technicians.

#### 2.3.4 Exercise capacity

The 6MWT was performed according to ATS guidelines (11) in a 30-meter corridor, recording 6MWD. Blood pressure, heart rate, and oxygen saturation were monitored before and after testing, with dyspnea assessed using the Borg scale.

Laboratory assessments

(1) Inflammatory markers: CRP was measured by immunoturbidimetry (Roche Cobas c702, reference: <5 mg/L); WBC count and neutrophil percentage were determined using the Sysmex XN-9000 analyzer. (2) Nutritional markers: albumin and total protein were measured by the bromocresol green method; BMI was calculated as weight (kg)/height^2^ (m^2^).

#### 2.3.5 Definition and calculation of the nutritional adequacy score

The nutritional adequacy score was a continuous variable (0–100) representing each patient’s average daily nutritional intake relative to individualized targets across the intervention period. Daily caloric and protein intake were recorded by registered dietitians using 24-h dietary recalls supplemented by hospital meal records. Individualized targets were derived following ESPEN guidelines (15): energy requirement was set at 25–30 kcal/kg/day for stable patients and 30–35 kcal/kg/day during exacerbation; protein requirement was set at 1.2–1.5 g/kg/day. The daily adequacy ratio was computed as [actual intake / individualized target] × 100, capped at 100 to prevent over-rewarding excess intake. The patient-level score reported in analyses was the time-weighted average of all available daily adequacy ratios over the intervention period (typically 24–28 weeks). A higher score indicates greater concordance between actual intake and prescribed nutritional targets.

Additional variables: Intervention details were recorded, including weekly training frequency, session duration, inspiratory muscle training intensity, and the nutritional adequacy score (as defined above).

### 2.4 Statistical analysis

Statistical analyses were performed using SPSS 26.0 (IBM Corp., Armonk, NY, USA) and R 4.2.0. Normality was assessed by the Shapiro–Wilk test. Normally distributed continuous variables were expressed as mean ± standard deviation, non-normally distributed variables as median (interquartile range), and categorical variables as frequency (percentage).

Pre-post comparisons were conducted using paired t-tests (for normally distributed data) or Wilcoxon signed-rank tests (for non-normally distributed data). Between-group comparisons employed independent-samples t-tests or Mann–Whitney U tests.

Stepwise multiple linear regression was used to explore potential predictors of ΔFEV1. Variables with *p* < 0.20 in univariate analysis were entered as candidates; the final model included age, sex, disease type, disease duration, baseline FEV1, baseline BMI, baseline albumin, weekly training sessions, session duration, IMT intensity, and nutritional adequacy score as pre-specified covariates. Given the exploratory nature of this analysis and the non-significant overall model fit, regression results are presented as hypothesis-generating rather than confirmatory findings. Multicollinearity among covariates was assessed using variance inflation factors (VIF; threshold > 5), and residual normality and homoscedasticity were verified by Q-Q plots and the Breusch–Pagan test (see Supplementary Figure S1). Correlations among change variables (ΔFEV1, ΔCRP, Δ6MWD, ΔBMI, ΔAlbumin) were assessed using Pearson coefficients and visualized as scatter plots and a correlation heatmap.

Sample size was estimated based on prior studies (18), assuming a mean FEV1 improvement of 0.15 L, a standard deviation of 0.20 L, *α* = 0.05, and 80% power, accounting for 10% attrition, yielding a minimum requirement of 88 patients. All tests were two-sided, with *p* < 0.05 considered statistically significant.

## 3 Results

### 3.1 Baseline characteristics

A total of 100 patients were enrolled, including 52 (52%) with COPD, 31 (31%) with bronchiectasis, and 17 (17%) with ILD. The mean age was 57.74 ± 8.81 years (range: 34–78 years), with 41 males (41%) and 59 females (59%). The median disease duration was 24.5 months (IQR: 12.0–48.5). A history of smoking was reported in 23 patients (23%), prior adenovirus infection in 18 (18%), and malnutrition in 27 (27%). Baseline characteristics are presented in Table 1.

**Table 1:** Baseline characteristics of the study population (*n* = 100).

Variable	Value
Age (years)	57.74 ± 8.81
Sex (male/female)	41/59
Disease type, *n* (%)	
COPD	52 (52%)
Bronchiectasis	31 (31%)
Interstitial lung disease	17 (17%)
Disease duration (months), median (IQR)	24.5 (12.0–48.5)
Smoking history, *n* (%)	23 (23%)
Prior adenovirus infection, *n* (%)	18 (18%)
History of malnutrition, *n* (%)	27 (27%)
Baseline BMI (kg/m^2^)	18.15 ± 2.40
Baseline FEV1 (L)	1.47 ± 0.43
Baseline FEV1 (% predicted)	52.3 ± 15.6
Baseline 6MWD (m)	428.97 ± 88.59
Baseline CRP (mg/L)	6.85 ± 4.50
Baseline WBC (×10^9^/L)	7.23 ± 1.95
Baseline neutrophil percentage (%)	61.70 ± 9.41
Baseline albumin (g/L)	39.12 ± 4.49
Baseline total protein (g/L)	67.11 ± 6.38

### 3.2 Changes in key parameters following intervention

After a mean intervention period of 6.8 ± 1.2 months, significant pre-post changes were observed across all assessed domains (Table 2).

**Table 2:** Comparison of key variables before and after intervention.

Variable	Pre-intervention	Post-intervention	Change	*p* value
Pulmonary function				
FEV1 (L)	1.47 ± 0.43	1.65 ± 0.44	0.18 ± 0.14	<0.001
FVC (L)	2.38 ± 0.65	2.59 ± 0.67	0.21 ± 0.18	<0.001
FEV1/FVC (%)	61.8 ± 8.9	63.7 ± 9.1	1.9 ± 4.2	0.003
Exercise tolerance				
6MWD (m)	428.97 ± 88.59	470.40 ± 92.99	41.43 ± 38.24	<0.001
Borg score	4.2 ± 1.3	3.1 ± 1.1	−1.1 ± 0.9	<0.001
Inflammatory markers				
CRP (mg/L)	6.85 ± 4.50	4.95 ± 4.71	−1.90 ± 2.01	<0.001
WBC (×10^9^/L)	7.23 ± 1.95	6.72 ± 2.06	−0.51 ± 1.39	<0.001
Neutrophil (%)	61.70 ± 9.41	58.91 ± 9.80	−2.79 ± 3.85	<0.001
Nutritional markers				
BMI (kg/m^2^)	18.15 ± 2.40	18.59 ± 2.47	0.44 ± 0.53	<0.001
Albumin (g/L)	39.12 ± 4.49	40.88 ± 4.77	1.76 ± 1.62	<0.001
Total protein (g/L)	67.11 ± 6.38	69.46 ± 6.54	2.35 ± 2.86	<0.001

#### 3.2.1 Pulmonary function

FEV1 increased from 1.47 ± 0.43 L to 1.65 ± 0.44 L, with a mean improvement of 0.18 ± 0.14 L (*p* < 0.001). Seventy-six percent of patients (76/100) reached or exceeded the COPD MCID for FEV1 of ≥100 mL (76% in COPD, 71% in bronchiectasis, and 82% in ILD subgroups). FEV1 percent predicted rose from 52.3 ± 15.6% to 58.9 ± 15.8% (*p* < 0.001). FVC increased from 2.38 ± 0.65 L to 2.59 ± 0.67 L (*p* < 0.001). The FEV1/FVC ratio improved from 61.8 ± 8.9% to 63.7 ± 9.1% (*p* = 0.003).

#### 3.2.2 Exercise tolerance

6MWD increased from 428.97 ± 88.59 m to 470.40 ± 92.99 m, with a mean improvement of 41.43 ± 38.24 m (*p* < 0.001), exceeding the minimal clinically important difference (MCID) of 30 m (19). Sixty-three percent of patients (63/100) achieved or exceeded the 30-m 6MWD MCID (60% in COPD, 65% in bronchiectasis, and 71% in ILD subgroups). The Borg dyspnea score decreased from 4.2 ± 1.3 to 3.1 ± 1.1 (*p* < 0.001).

#### 3.2.3 Inflammatory markers

CRP declined from 6.85 ± 4.50 mg/L to 4.95 ± 4.71 mg/L (*p* < 0.001), representing a 27.7% reduction. WBC decreased from 7.23 ± 1.95 to 6.72 ± 2.06 × 10^9^/L (*p* < 0.001). Neutrophil percentage fell from 61.70 ± 9.41% to 58.91 ± 9.80% (*p* < 0.001).

#### 3.2.4 Nutritional status

BMI increased from 18.15 ± 2.40 to 18.59 ± 2.47 kg/m^2^ (*p* < 0.001). Serum albumin rose from 39.12 ± 4.49 to 40.88 ± 4.77 g/L (*p* < 0.001). Total protein increased from 67.11 ± 6.38 to 69.46 ± 6.54 g/L (*p* < 0.001).

### 3.3 Exploratory analysis: predictors of FEV₁ improvement

Multiple linear regression analysis (Table 3), after adjusting for age, sex, disease type, disease duration, and baseline FEV1, did not identify any statistically significant independent predictor of ΔFEV1. The overall model failed to reach significance (R^2^ = 0.142, adjusted R^2^ = 0.038, *F*(12, 87) = 1.20, *p* = 0.297), indicating that the included variables collectively explained only a modest proportion of variance. These results are therefore interpreted as exploratory and hypothesis-generating rather than confirmatory (Figure 1).

**Table 3:** Multiple linear regression analysis of ΔFEV₁ (exploratory).

Variable	*β* coefficient	95% CI	*P* value
Age	0.001	−0.001 to 0.004	0.274
Male sex	0.044	−0.014 to 0.103	0.139
Disease duration (months)	−0.001	−0.003 to 0.002	0.613
Prior adenovirus infection	0.005	−0.061 to 0.071	0.882
History of malnutrition	0.023	−0.040 to 0.086	0.478
Baseline BMI	−0.003	−0.015 to 0.009	0.647
Baseline albumin	0.002	−0.005 to 0.008	0.607
Baseline FEV1	−0.031	−0.096 to 0.034	0.348
Weekly training sessions	0.011	−0.015 to 0.036	0.408
Session duration (min)	0.001	−0.003 to 0.005	0.666
IMT intensity	0.079	−0.127 to 0.285	0.455
Nutritional adequacy score	0.024	−0.004 to 0.052	0.093

R^2^ = 0.142, adjusted R^2^ = 0.038, F(12, 87) = 1.20, *p* = 0.297. IMT, inspiratory muscle training. This model did not reach overall statistical significance and results should be considered exploratory. Full residual diagnostics, VIF values, and a sensitivity model excluding history of malnutrition are provided in Supplementary Figure S1.

**Figure 1:**
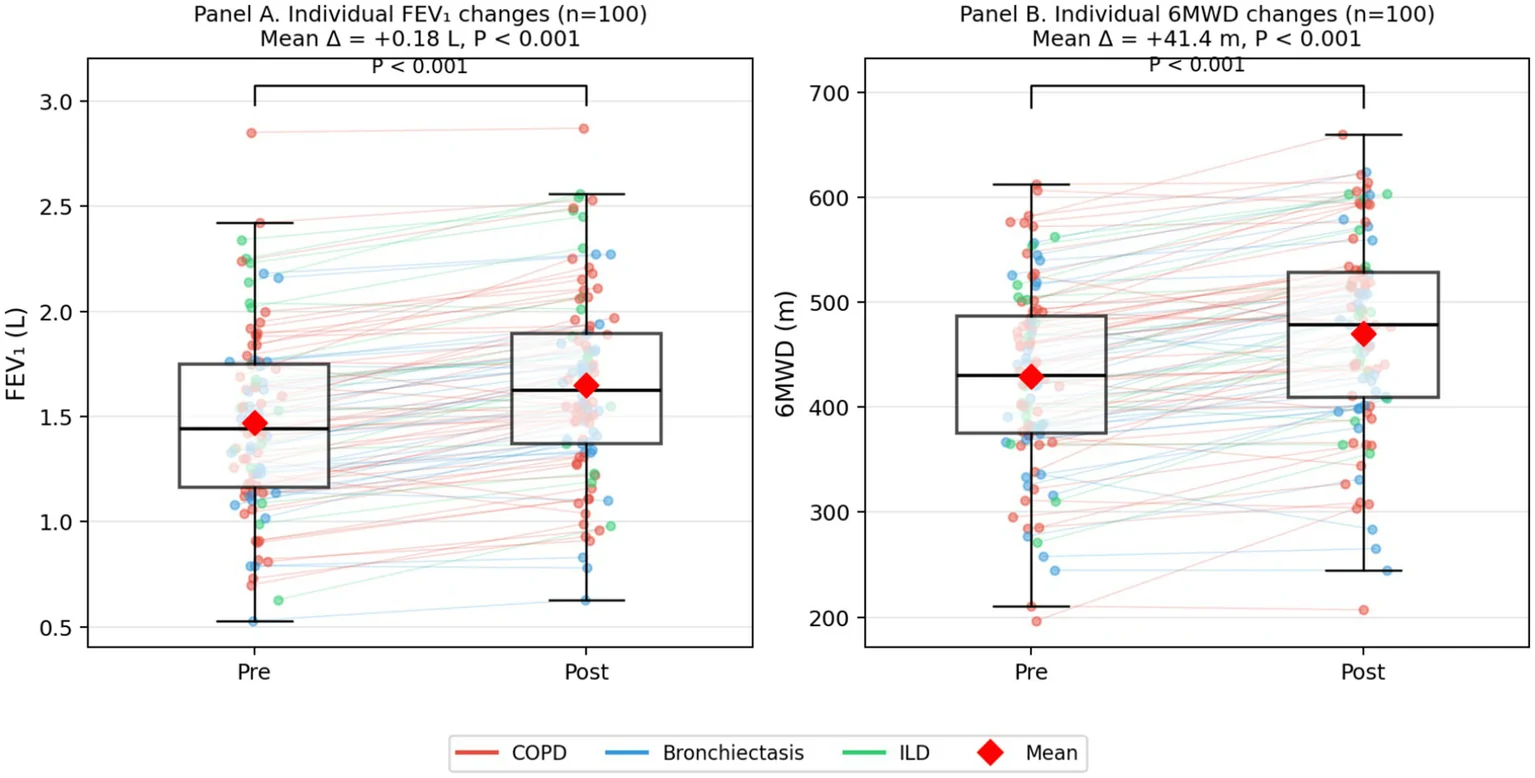
Paired individual-level changes in FEV₁ **(A)** and 6MWD **(B)**. Each thin colored line represents one patient connecting the patient’s pre- and post-intervention measurements; line color denotes disease type (red = COPD, blue = Bronchiectasis, green = ILD). Overlaid boxplots show median and IQR; red diamonds indicate group means. Both changes were statistically significant (paired t-test, *p* < 0.001). This visualization replaces the previous summary-only boxplot to show individual treatment-associated trajectories per Reviewer 2’s suggestion.

The nutritional adequacy score showed a non-significant positive trend (*β* = 0.024, 95% CI: −0.004 to 0.052, *p* = 0.093), which may warrant investigation in adequately powered prospective studies. Similarly, male sex (*β* = 0.044, *p* = 0.139) and IMT intensity (*β* = 0.079, *p* = 0.455) showed directional but non-significant trends. The absence of significant predictors likely reflects the small sample size, heterogeneous disease population, and unmeasured confounders rather than a true null relationship. Diagnostic checks indicated no problematic multicollinearity (all VIF < 2.5), and residuals were approximately normal with constant variance (Breusch–Pagan *p* = 0.21). Full model diagnostics are provided in Supplementary Figure S1.

### 3.4 Subgroup analysis

By sex (Table 4), males demonstrated slightly greater FEV1 improvement (0.21 ± 0.13 L) compared with females (0.17 ± 0.15 L), without reaching statistical significance (*p* = 0.121). Males showed significantly greater 6MWD improvement (48.5 ± 40.2 m) than females (36.5 ± 36.1 m, *p* = 0.045).

**Table 4:** Subgroup analysis of key changes by sex.

Variable	Male (*n* = 41)	Female (*n* = 59)	*P* value
ΔFEV1 (L)	0.21 ± 0.13	0.17 ± 0.15	0.121
Δ6MWD (m)	48.5 ± 40.2	36.5 ± 36.1	0.045
ΔBMI (kg/m^2^)	0.42 ± 0.51	0.45 ± 0.54	0.734
ΔCRP (mg/L)	−2.1 ± 2.2	−1.8 ± 1.9	0.389
ΔAlbumin (g/L)	1.8 ± 1.7	1.7 ± 1.6	0.812

When stratified by disease type (Table 5), ILD patients exhibited the largest FEV1 improvement (0.22 ± 0.14 L), followed by COPD (0.18 ± 0.15 L) and bronchiectasis (0.17 ± 0.11 L), though differences were not statistically significant (one-way ANOVA, *p* = 0.535). These non-significant inter-group differences should be interpreted cautiously given the sample sizes per subgroup.

**Table 5:** Subgroup analysis of ΔFEV₁ by disease type.

Disease type	*n*	ΔFEV1 (L), mean ± SD	*P* value (ANOVA)
COPD	52	0.18 ± 0.15	0.535
Bronchiectasis	31	0.17 ± 0.11	
ILD	17	0.22 ± 0.14	

By baseline BMI (<18.5 vs. ≥ 18.5 kg/m^2^), the underweight group showed comparable FEV1 improvement (0.19 ± 0.14 L vs. 0.17 ± 0.13 L, *p* = 0.421), but significantly greater BMI gain (0.58 ± 0.55 vs. 0.31 ± 0.48 kg/m^2^, *p* = 0.012).

### 3.5 Correlation analysis and new visualizations

Pearson correlation analysis revealed the following key associations: (1) ΔFEV1 showed a moderate positive correlation with Δ6MWD (*r* = 0.41, *p* < 0.001), indicating that patients with greater pulmonary function gains also tended to improve more in exercise capacity (Figure 2). (2) ΔFEV1 showed a significant negative correlation with ΔCRP (*r* = −0.21, *p* = 0.039), suggesting that greater reductions in systemic inflammation co-occurred with larger FEV1 improvements (Figure 3). (3) ΔFEV1 was weakly and non-significantly correlated with ΔBMI (*r* = 0.10, *p* = 0.347), indicating that nutritional weight changes were not a strong correlate of pulmonary function gains. (4) A correlation heatmap across all change variables is presented in Figure 4, demonstrating that the FEV1–6MWD and FEV1–CRP associations were the most prominent relationships (Table 6).

**Figure 2:**
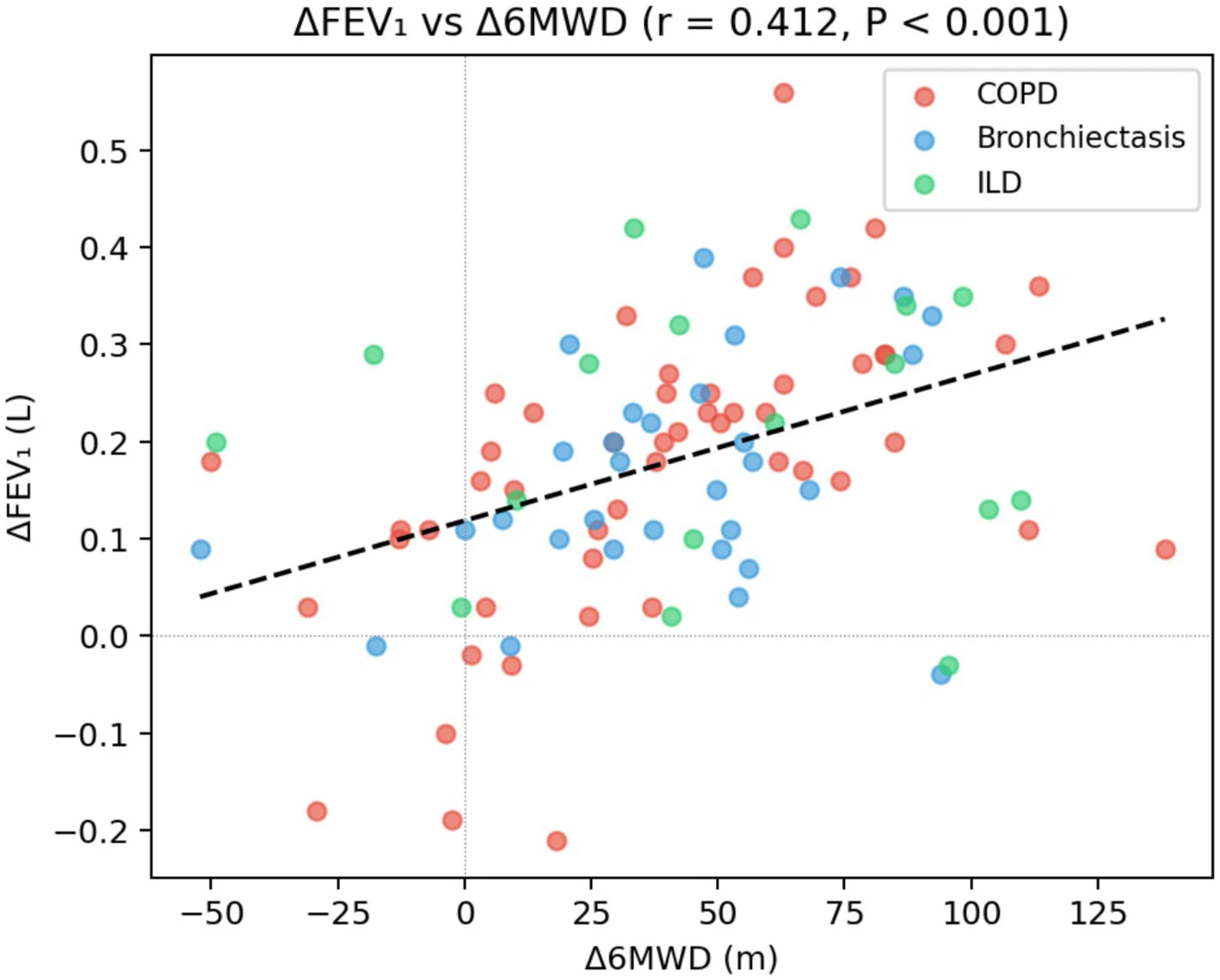
Scatter plot of ΔFEV₁ versus Δ6MWD. Each point represents one patient, colored by disease type. Regression line shown; *r* = 0.41, *p* < 0.001.

**Figure 3:**
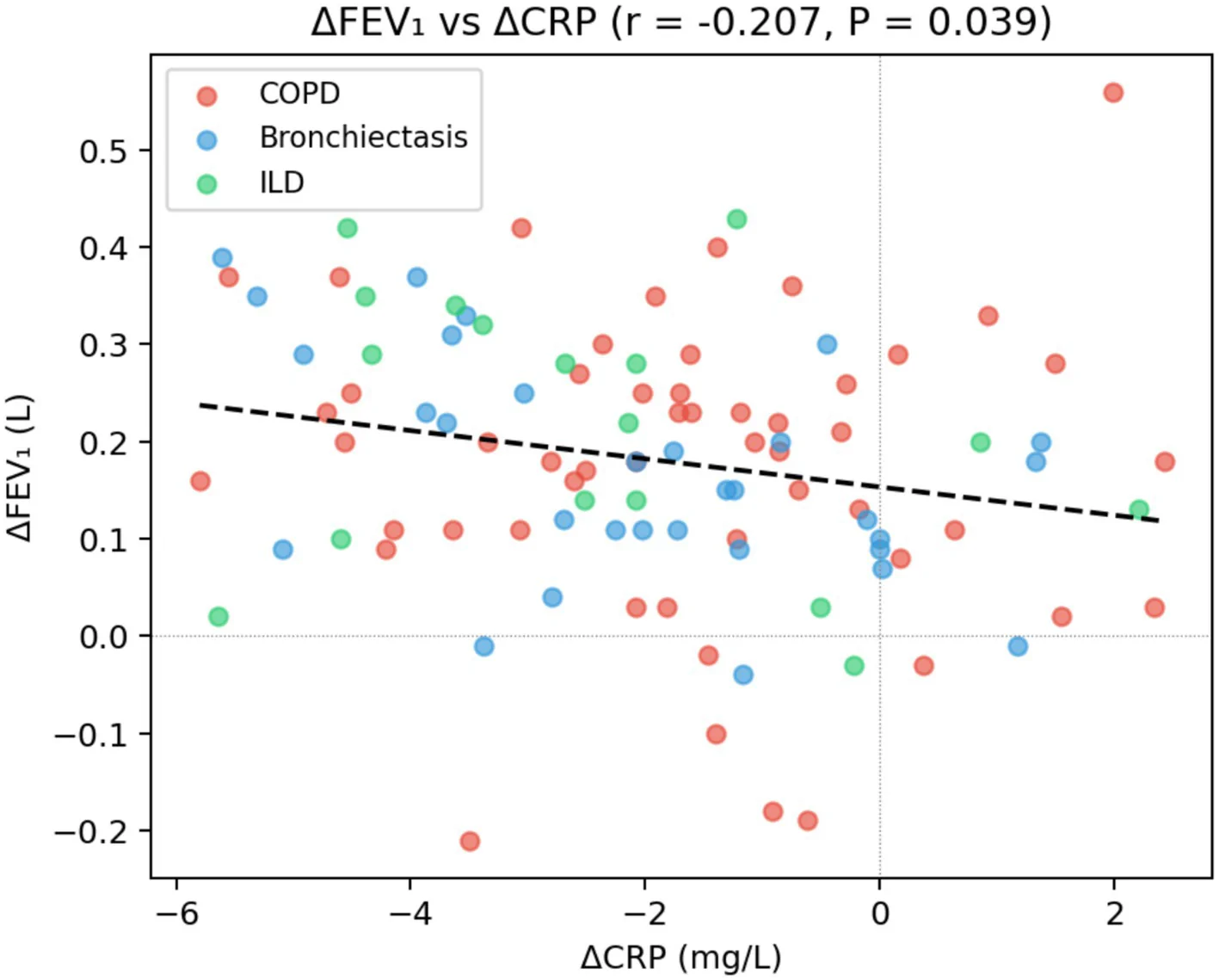
Scatter plot of ΔFEV₁ versus ΔCRP. Each point represents one patient, colored by disease type. Regression line shown; *r* = −0.21, *p* = 0.039.

**Figure 4:**
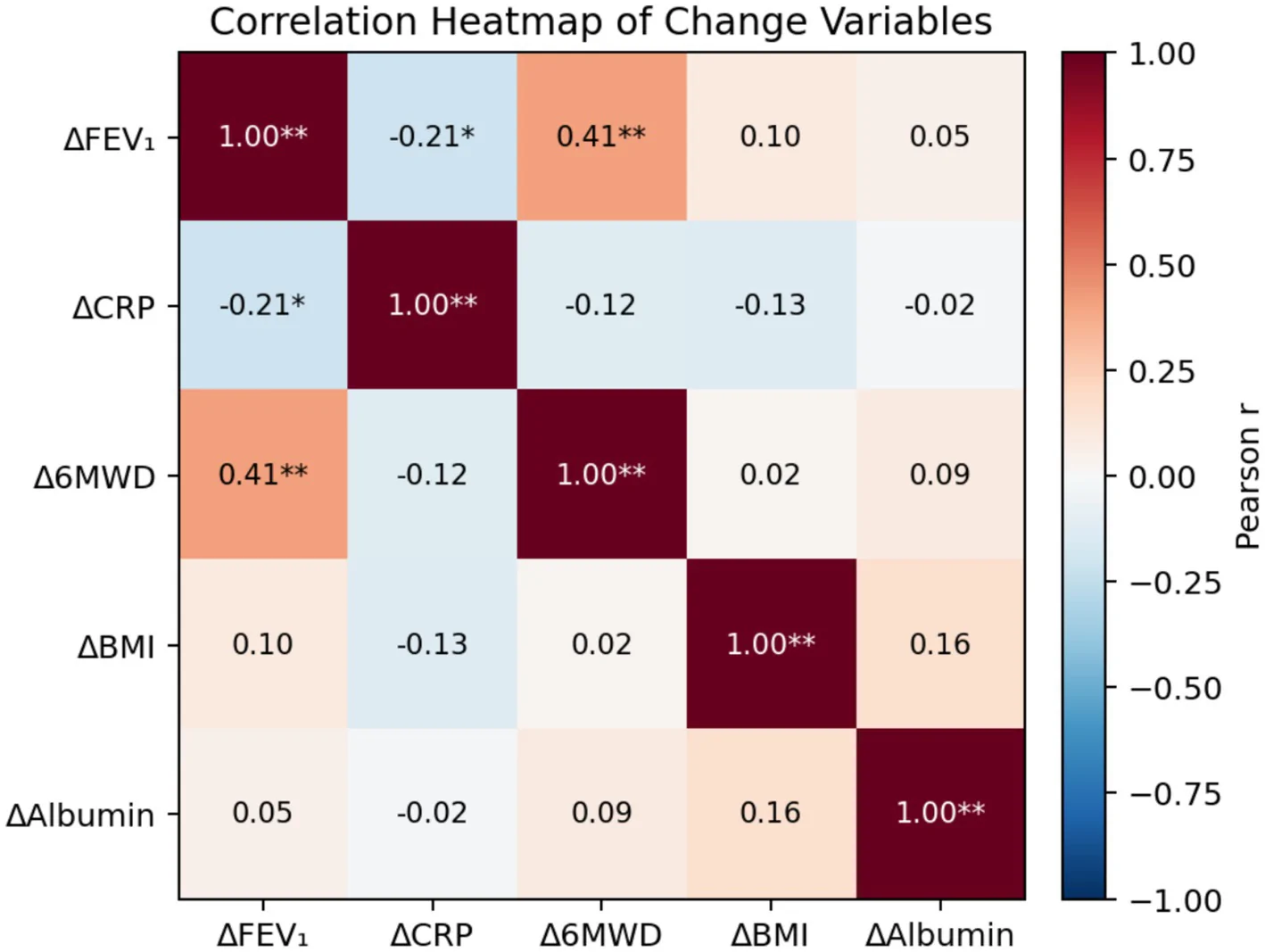
Correlation heatmap of change variables (ΔFEV₁, ΔCRP, Δ6MWD, ΔBMI, ΔAlbumin). Color scale represents Pearson *r*; * *p* < 0.05, **p < 0.001.

**Table 6:** Pearson correlation matrix of change variables.

Variable	ΔFEV1	ΔCRP	Δ6MWD	ΔBMI	ΔAlbumin
ΔFEV1	1.000	−0.207*	0.412**	0.095	0.103
ΔCRP	−0.207*	1.000	−0.118	−0.052	−0.041
Δ6MWD	0.412**	−0.118	1.000	0.081	0.059
ΔBMI	0.095	−0.052	0.081	1.000	0.149
ΔAlbumin	0.103	−0.041	0.059	0.149	1.000

*P < 0.05; **P < 0.001.

### 3.6 Safety assessment

No serious adverse events were observed during the intervention period. Mild musculoskeletal soreness occurred in 12 patients (12%), and transient fatigue exacerbation in 8 (8%), both of which resolved after training intensity adjustment. Five patients (5%) temporarily suspended training for 1–2 weeks due to acute exacerbation, resuming after recovery. No patient withdrew due to adverse events.

## 4 Discussion

This retrospective study of 100 patients with chronic respiratory diseases evaluated pre- and post-intervention changes in pulmonary function, inflammatory status, and nutritional parameters, and explored associations among these domains. Given the uncontrolled, single-arm retrospective design, the observed pre-post changes represent associated patterns rather than established intervention effects; the following discussion should be read with this limitation in mind. Following approximately 6.8 months of integrated management, significant pre-post improvements were observed across all assessed domains.

### 4.1 Pulmonary function improvement and clinical relevance

The mean FEV1 improvement of 0.18 L (12.2%) exceeded the MCID of 100 mL for COPD patients (20). Importantly, beyond the mean-level threshold, 76% of patients individually achieved or exceeded the 100-mL FEV1 MCID, with achievement rates of 77, 71, and 82% in the COPD, bronchiectasis, and ILD subgroups, respectively. The fact that more than three-quarters of patients reached a clinically meaningful improvement supports the clinical—not merely statistical—relevance of the observed change. This finding is consistent with recent evidence. Holland et al. (14) demonstrated that pulmonary rehabilitation can increase FEV1 by 0.10–0.20 L in COPD, and Jenkins et al. (21) reported FEV1 improvements of approximately 0.15 L following comprehensive rehabilitation. The improvement observed in our cohort is consistent with published benchmarks and likely reflects the synergistic effects of multi-component intervention, comparatively preserved baseline lung function (FEV1 52.3% predicted), and adequate patient compliance.

However, it bears emphasis that the absence of a randomized control arm makes it impossible to attribute the observed FEV1 change solely to the intervention. Natural disease fluctuation, regression to the mean, and Hawthorne effects may have contributed to the observed pre-post differences.

### 4.2 Inflammation control and pulmonary function

We observed a 27.7% reduction in CRP and a significant negative correlation between ΔFEV1 and ΔCRP (*r* = −0.21, *p* = 0.039; Figure 3), indicating that patients who experienced greater decrements in systemic inflammation also tended to show larger improvements in lung function. This is the most methodologically robust associative finding in this study, given the formal significance threshold was met. This aligns with current understanding that systemic inflammation not only reflects pulmonary damage but actively drives disease progression (22).

CRP, an acute-phase protein induced by interleukin-6, has been linked to exacerbation risk, hospitalization, and mortality in COPD. Acute respiratory viral infections, including COVID-19, are increasingly recognized as potent triggers of systemic inflammatory activation in patients with chronic respiratory disease (23), and the recently refined ROME framework for COPD exacerbations highlights the prognostic importance of monitoring inflammatory severity during such events (24). Celli et al. (25) demonstrated that patients with persistently elevated CRP experienced accelerated FEV1 decline compared with those maintaining lower inflammatory burden. In our study, CRP decreased from 6.85 to 4.95 mg/L, approaching a lower inflammatory state. The concomitant reduction in WBC count and neutrophil percentage further corroborates inflammatory amelioration.

### 4.3 Exercise capacity and the ΔFEV₁–Δ6MWD relationship

The 6MWD improvement of 41.43 m exceeded the MCID of 30 m and, most notably, showed a moderate positive correlation with ΔFEV1 (*r* = 0.41, *p* < 0.001; Figure 2). At the individual level, 63% of patients (60% COPD, 65% bronchiectasis, and 71% ILD) achieved the 30-m MCID threshold. Using the 25-m lower bound of MCID estimates reported by Holland et al. (19), the responder rate rose to 69%. The convergence of group-level and individual-level evidence reinforces the clinical relevance of the change. This is the strongest bivariate association identified in this study and suggests that pulmonary function and exercise capacity co-improve in a coordinated fashion, consistent with the integrated nature of the cardiopulmonary–musculoskeletal axis.

Multiple mechanisms underlie this improvement: aerobic training enhances mitochondrial density and oxidative enzyme activity in skeletal muscle, improving oxygen utilization; it also reduces ventilatory demand and dynamic hyperinflation. Additionally, exercise exerts anti-inflammatory effects by reducing circulating interleukin-6 and tumor necrosis factor-*α* (26). The sex-based difference in 6MWD improvement (males 48.5 m vs. females 36.5 m, *p* = 0.045) may reflect differences in baseline physical fitness, muscle mass, and training responsiveness.

### 4.4 Impaired nutritional status: modest changes, exploratory associations

Nutritional improvement was observed across all markers (ΔBMI = +0.44 kg/m^2^, ΔAlbumin = +1.76 g/L). However, the regression model did not identify the nutritional adequacy score as a statistically significant predictor of ΔFEV1 (*p* = 0.093), and the correlation between ΔFEV1 and ΔBMI was weak and non-significant (*r* = 0.10, *p* = 0.347). Medical nutrition therapy has gained increasing recognition as a complementary component of COPD management (27), although randomized evidence specifically in malnourished outpatients remains limited and indicates only modest functional gains (28). These results are consistent with the broader literature’s mixed findings regarding short-term nutritional intervention in patients with impaired nutritional status and lung function. The trend-level association of nutritional adequacy score (*p* = 0.093) may warrant prospective investigation but cannot be interpreted as evidence of effect in the current design. Over-interpreting weak nutritional associations risks misallocating clinical resources.

### 4.5 Implications of disease heterogeneity for the observed associations

Our cohort comprised three diseases (COPD 52%, bronchiectasis 31%, ILD 17%) with substantially different pathophysiological mechanisms, which deserves careful consideration when interpreting the observed associations. COPD is characterized by progressive, largely irreversible airflow obstruction driven by chronic airway inflammation and emphysematous parenchymal destruction; consequently, FEV1 improvements in COPD typically reflect amelioration of bronchospasm, reduced air trapping, and improved respiratory mechanics rather than reversal of structural damage. Bronchiectasis, by contrast, is dominated by chronic airway dilation with mucus retention and recurrent infection, such that improvements may be more sensitive to airway clearance, antimicrobial therapy, and resolution of exacerbations. ILD involves progressive parenchymal fibrosis and a restrictive ventilatory defect, where short-term FEV1 changes more often reflect resolution of subacute inflammation or improvement in inspiratory muscle function than reversal of fibrotic remodeling. Given these mechanistic differences, the moderate positive ΔFEV1–Δ6MWD association we observed is plausibly more robust than the modest ΔFEV1–ΔCRP association, because the former reflects a generic cardiopulmonary–musculoskeletal coupling shared across diseases, whereas systemic inflammation may play distinctly different roles—particularly in stable ILD where neutrophilic inflammation is less central. Disease heterogeneity may therefore dilute disease-specific signals in pooled analyses and partially explain why the regression model did not reach overall significance. Disease-stratified subgroup point estimates were directionally similar but underpowered to detect interactions. Adequately sized, disease-specific cohorts will be needed to clarify whether the pooled associations represent a true shared mechanism or an averaging artifact.

### 4.6 Advantages of comprehensive management

The integrated approach combining pharmacotherapy, rehabilitation, nutritional support, and health education reflects the holistic paradigm of modern respiratory disease management. Compared with single-modality intervention, comprehensive strategies offer synergistic benefits. Rochester et al. (29) reported that comprehensive programs yielded greater FEV1 improvement than pharmacotherapy alone or rehabilitation alone. The emphasis on individualized treatment likely contributed to the favorable observed outcomes (30).

### 4.7 Limitations and future directions

This study has several important limitations that constrain interpretability. First, the retrospective, single-arm design precludes causal inference; observed pre-post changes cannot be attributed to the intervention without a concurrent control group. In particular, several non-intervention factors plausibly contributed to the observed improvements and must be considered when interpreting our findings: (i) medication adjustments during follow-up (optimization of inhaled therapy, antibiotic courses for exacerbations, or initiation of long-acting bronchodilators) may have independently improved FEV1 and 6MWD; (ii) natural recovery from acute exacerbations near baseline could artificially elevate post-intervention values, particularly for inflammatory markers (CRP, WBC); (iii) regression to the mean is a real concern because patients are typically enrolled at relatively impaired clinical status, and statistical drift toward the population mean at follow-up would mimic an intervention effect; and (iv) test learning effects and increased familiarity with repeated 6MWT and spirometry maneuvers can yield apparent improvements of 5–20 m in 6MWD and small FEV1 gains independent of any physiological change. The magnitude of each contribution cannot be parsed in our design, and we acknowledge that the observed pre-post changes likely reflect a composite of intervention-related and intervention-independent effects. Second, the single-center setting limits generalizability. Third, the relatively small sample size reduced statistical power for regression modeling (overall model *p* = 0.297) and subgroup analyses. Fourth, the absence of long-term follow-up prevents evaluation of the durability of observed improvements. Fifth, the heterogeneous disease composition (COPD 52%, bronchiectasis 31%, ILD 17%) with different underlying pathophysiologies may dilute disease-specific signals in pooled analyses (see Section 4.5). Sixth, confounding by disease severity, comorbidity burden, socioeconomic status, medication adherence, and baseline activity levels cannot be fully accounted for in the retrospective design.

Future research directions include: (1) multi-center prospective randomized controlled trials with active comparators and parallel non-rehabilitation control arms to isolate the intervention effect from concurrent medication changes and natural recovery; (2) longer follow-up to evaluate outcome durability; (3) disease-stratified analyses with adequate per-group sample sizes designed to test mechanistic differences across COPD, bronchiectasis, and ILD; and (4) biomarker-guided personalized rehabilitation strategies.

### 4.8 Clinical implications

Despite the design limitations, these findings are consistent with the value of comprehensive, multidimensional assessment routinely monitoring pulmonary function, inflammatory markers, and nutritional status. They support multidisciplinary collaboration integrating respiratory, rehabilitation, and nutritional expertise, and highlight the value of early, individualized intervention strategies.

## 5 Conclusion

This retrospective analysis of 100 patients with chronic respiratory diseases demonstrated significant pre-post changes in pulmonary function, exercise tolerance, and nutritional status alongside reductions in systemic inflammation following comprehensive intervention. Notably, improvements in FEV1 were significantly associated with improvements in both 6MWD (*r* = 0.41, *p* < 0.001) and reductions in CRP (*r* = −0.21, *p* = 0.039), providing exploratory evidence of inter-domain relationships. However, no independent predictor of ΔFEV1 was identified in regression analysis, and causal conclusions cannot be drawn from this uncontrolled design. These findings support multidimensional, individualized management strategies. Future prospective controlled trials are warranted to establish efficacy and to determine the optimal combination of intervention components.

## Data Availability

The original contributions presented in the study are included in the article/Supplementary material, further inquiries can be directed to the corresponding author.
